# The simplified GLIM criteria for assessment of malnutrition and its correlation with clinical outcomes in Crohn’s disease patients

**DOI:** 10.3389/fnut.2024.1414124

**Published:** 2025-01-07

**Authors:** Xiaomei Song, Xiaoxin Zhou, Hao Wang, Hong Guo, Jian Yang

**Affiliations:** ^1^Research Center for Metabolic and Cardiovascular Diseases, The Third Affiliated Hospital of Chongqing Medical University, Chongqing, China; ^2^Department of Clinical Nutrition, The Third Affiliated Hospital of Chongqing Medical University, Chongqing, China; ^3^Department of Gastroenterology, Chongqing General Hospital, Chongqing University, Chongqing, China

**Keywords:** Crohn’s disease, GLIM criteria, malnutrition, clinical outcome, endoscopic remission

## Abstract

**Objective:**

Patients with Crohn’s disease (CD) commonly experience malnutrition. The Global Leadership Initiative on Malnutrition (GLIM) criteria, a novel approach to assessing malnutrition, has been validated in some diseases. However, there are limited studies in CD patients. This study aimed to investigate the applicability and effectiveness of the simplified GLIM criteria for evaluating the nutritional status of patients with Crohn’s disease. Additionally, it sought to evaluate the correlation between malnutrition defined by simplified GLIM and clinical outcomes.

**Methods:**

A retrospective cohort study was conducted with 386 patients with CD. Data were extracted from the medical records, including demographic and clinical characteristics. All patients were evaluated using the simplified GLIM criteria. The prevalence of malnutrition was reported and the relationship between malnutrition and clinical outcome was analyzed.

**Results:**

The prevalence of malnutrition among patients with CD was 73.6%, with 36.5% classified as moderate malnutrition and 37.0% classified as severe malnutrition. The malnourished group had significantly higher Crohn’s Disease Activity Index (CDAI) scores compared to the non-malnourished group (*p* < 0.001). Furthermore, the malnutrition group exhibited significantly lower levels of specific nutritional indicators, including hemoglobin (*p* = 0.040), albumin (*p* = 0.015), and prealbumin (*p* = 0.021). The median duration of follow-up in the cohort was 15.2 weeks. The results indicated that malnutrition, as assessed by simplified GLIM, independently influenced endoscopic remission (*p* = 0.033). Additionally, the duration of disease (*p* = 0.021), C-reactive protein (*p* = 0.014) and prealbumin (*p* = 0.014) were independent factors influencing endoscopic remission in patients with CD.

**Conclusion:**

Malnutrition identified using the simplified GLIM criteria is associated with age, CDAI, behavior, hemoglobin, and albumin, providing prognostic value for endoscopic remission in CD patients.

## Introduction

Inflammatory bowel disease (IBD), a chronic and non-specific inflammatory condition, impacts the entire gastrointestinal tract. Its prevalence is steadily rising in both developed and developing countries (1). Patients with IBD commonly experience malnutrition issues (2). Malnutrition in IBD patients results from various complex factors such as inflammatory response and clinical complications, subsequently leading to reduced intake, nutrient loss, and malabsorption (2, 3). Importantly, malnutrition further causes increased occurrence of complications, hospitalization, and surgery. Therefore, it is crucial to conduct malnutrition assessment in IBD patients.

Currently, there are no standardized evaluation tools available for patients with IBD. Other nutritional assessment tools include: (1) Subjective global assessment (SGA), a subjective method that combines clinical evaluation and medical history to accurately reflect the nutritional status of patients (4); (2) Patient-generated subjective global assessment (PG-SGA), which is more used in cancer patients (5); (3) Mini nutritional assessment (MNA), which is primarily for elderly patients but is also applicable for assessing nutritional status in IBD patients (6). In 2018, the Global Leadership Initiative on Malnutrition (GLIM) criteria for diagnosis of malnutrition was issued, and then has attracted more and more attentions in recent years (7). The aim of implementing the GLIM criteria is to establish a unified approach for diagnosing malnutrition and improving the identification of malnutrition.

Up to now, the GLIM criteria has been validated in various diseases (8–10). However, it has not been widely used in patients with IBD. There are only a few studies reporting the application of GLIM criteria in IBD patients (11–14). For example, the study by Huang et al. showed that the prevalence of malnutrition among IBD patients, as determined by the GLIM criteria, was 41.5% in a preoperative evaluation of 53 cases; this rate was higher than the malnutrition rate of 27.0% assessed by the criteria of ESPEN 2015 (15). Another study suggested a significant association between malnutrition diagnosed by GLIM in ulcerative colitis (UC) patients and opportunistic infections, as well as a trend toward surgical treatment, which significantly increased the risk of readmission (13). It is evident that further research is required to investigate the application of GLIM criteria in IBD. It should be noted that a few studies in China excluded the muscle-related indicator from GLIM criteria for various reasons (16). Therefore, we referred to this as the simplified GLIM criteria in the subsequent text.

Our present study aimed to explore the application of simplified GLIM criteria in evaluating malnutrition among Chinese patients with Crohn’s disease (CD) and identify the risk factors associated with malnutrition in a large sample of CD patients. These will improve our understanding of the impact of malnutrition on the prognosis of CD patients and provide more precise nutritional intervention recommendations for clinical practice.

## Subjects and methods

### Study populations

**Figure 1:**
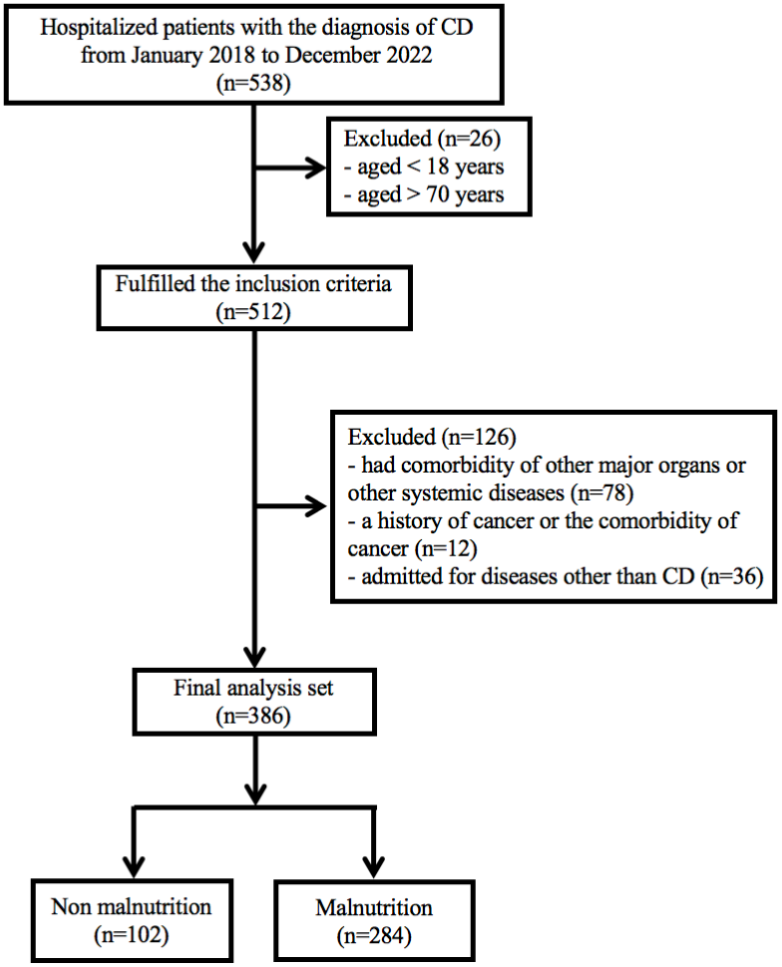
Flowchart shows inclusion and exclusion criteria for patients in the study.

In recent years, biologics have emerged as the primary treatment for patients with Crohn’s disease (17, 18). This is a retrospective study conducted at Chongqing General Hospital (Chongqing, China), which included CD patients who received the treatment with biologics. The study period spanned from January 1, 2018 to December 31, 2022. The inclusion criteria for the study were as follows: (1) patients aged over 18 years and under 70 years; (2) a confirmed diagnosis of CD for at least 3 months. The diagnosis of CD was based on clinical manifestations, endoscopic evidence, and histopathological reports, following the guidelines outlined in the 2018 Consensus on Diagnosis and Treatment of inflammatory bowel disease (19); (3) only CD patients who had used or were currently using biologics were included. The exclusion criteria include: (1) patients had comorbidity of other major organs or other systemic diseases; (2) a history of cancer or the comorbidity of cancer; (3) admitted for diseases other than CD. Participants were followed up from their initial admission until the end of December, 2022. Patients with missing follow-up data or other essential information were excluded, as indicated in the study protocol (Figure 1). Ultimately, a total of 386 patients were included in the current study. The study has been approved by the Medical Ethics Committee of Chongqing General Hospital (approval number: KY S2022-023-01). All procedures of this study met the basic ethical standards required by the Declaration of Helsinki. Data of this study were retrieved through our IBD database. All patients provided written informed consent.

### Data collection

From January 2018 to December 2022, all patients were consecutively enrolled and their nutritional status was assessed by a trained dietician. General medical history, anthropometric measurements, laboratory results, co-morbidity information, and nutrition-related data were collected. The assessment of disease activity was based on the Crohn’s Disease Activity Index (CDAI): the CDAI score < 150 was considered to indicate disease remission, while CDAI score ≥ 150 was defined as disease activity (20). The Simplified Endoscopic Score for CD (SES-CD) was used as an assessment indicator after endoscopic examinations. Endoscopic remission is defined as SES-CD scores ranging from 0 to 3 (21). In this study, data from each patient’s initial visit to our hospital served as the basis for analysis. We selected the results from the initial follow-up endoscopy conducted at our hospital to assess endoscopic remission. Disease phenotype was categorized as L1-L3, and B1-B3, according to the Montreal classification (22). Information of the treatment and follow-up of CD patients was also recorded. During the initial year of the treatment, each patient received an endoscopic examination, and the relevant biochemical markers were also assessed. Patients followed up for more than one year were asked about hospital admissions for assessing the recurrence, necessity of surgery, and other changes.

### Assessment of nutritional status

The Nutritional Risk Screening 2002 (NRS-2002) was performed and patients with a score of 3 or higher were considered at nutritional risk (23). The simplified GLIM criteria were used to diagnosing malnutrition and assessing its severity scale. The simplified GLIM criteria consist of three phenotypic criteria and two etiological criteria. A combination of at least one phenotypic criterion and one etiological criterion is necessary for the diagnosis of malnutrition (23). For the phenotypic criteria, involuntary weight loss was assessed (24). The body mass index (BMI), calculated as weight (kg) divided by height squared (m^2^), is determined using a scale with precision to 0.1 kg and 0.01 m. The loss of muscle mass was not analyzed due to the absence of data collected in the original database. Regarding the etiological criteria, due to the location of the lesions primarily in the gastrointestinal system and the chronic and recurrent nature of IBD, all patients with clinical activity met the criteria for reduced food intake or digestion (25). Disease-associated inflammation is defined by C-reactive protein (CRP) level greater than 5 mg/L based on previous studies (26). This study did not analyze the relevant data for the third phenotype criterion, which is reduced muscle mass, due to the lack of data in the original database and the absence of unified body measurement standards between the Chinese and American populations. A similar issue was also identified in previously published papers, where the authors also failed to assess muscle loss data (27, 28). This oversight may introduce bias.

The severity scale of malnutrition was defined as stage I (moderate) and stage II (severe) malnutrition, assessed by the phenotypic scale (23, 24). Meeting any of the following criteria for weight loss would lead to a diagnosis of stage I (moderate malnutrition): a weight loss of 5–10% within the past six months or > 10–20% beyond six months, with a BMI <18.5 kg/m^2^ if <70 years or < 20 kg/m^2^ if >70 years. Stage II (severe malnutrition) is diagnosed when the weight loss criteria are >10% within the past six months or > 20% beyond six months, or with a low BMI of <17 kg/m^2^ if <70 years and < 17.8 kg/m^2^ if >70 years.

### Statistical analysis

The demographic and clinical parameters were reported as the mean ± standard deviation (SD) or median (Q1, Q3). Intergroup comparisons were conducted using the independent *t*-test or χ^2^ test. Before constructing the multivariate logistic regression model, we conducted a univariate analysis to identify potential variables associated with the research outcomes. We calculated the *p*-values for each variable and filtered out those with statistical significance (*p* < 0.05) to identify candidate variables for further analysis using SPSS version 22.0 (SPSS Inc., Chicago, Illinois, USA) for statistical analysis. Furthermore, we also consulted relevant literature and clinical experience to incorporate potentially significant variables.

## Results

### Demographics and clinical characteristics

A total of 538 hospitalized patients with a diagnosis of CD were included in this study. Finally, 386 CD patients were considered for the final analysis (Figure 1). Table 1 showed a summary of participants’ demographics and clinical features. Of the patients, 266 (68.9%) were male, with an average age of 29.8 ± 11.2 years old. The means of BMI and CDAI were 18.9 ± 3.8 kg/m^2^ and 111.5 ± 71.1, respectively. The age at diagnosis and duration of CD were 25.8 ± 10.12 years and 2.4 ± 2.9 years old, respectively. 80 (20.8%) patients had a history of smoking; 100 patients (25.9%) had a history of intestinal surgery. The primary disease location in CD patients was the ileocolonic region (246, 66.8%), and the most common disease behavior was B1. In addition, 208 (53.9%) patients had perianal lesions.

**Table 1:** Demographic and clinical characteristics of patients with or without malnutrition assessed by the GLIM criteria.

Variables	Non-malnourished *n* = 102 (26.4%)	Malnourished *n* = 284 (73.6%)	Total *n* = 386 (100%)	*p*
Age at assessment, years	33.9 ± 12.5	28.3 ± 10.3	29.8 ± 11.2	**<0.001**
Onset age, years	24.9 ± 12.6	21.6 ± 9.2	22.4 ± 10.3	**0.024**
Age at diagnosis, years	29.5 ± 11.8	24.7 ± 9.3	25.8 ± 10.2	**0.001**
Duration of disease, years	2.4 ± 2.8	2.4 ± 2.9	2.4 ± 2.9	0.984
Male, *n* (%)	76 (74.5%)	190 (66.9%)	266 (68.9%)	0.142
BMI (kg/m^2^)	22.4 ± 2.5	18.2 ± 2.3	18.9 ± 3.8	**<0.001**
CDAI	77.3 ± 61.3	122.9 ± 70.5	111.5 ± 71.1	**<0.001**
Smoking history, *n* (%)	23 (22.5%)	57 (20.1%)	80 (20.8%)	0.608
History of intestinal surgery (%)	26 (25.5%)	74 (26.1%)	100 (25.9%)	0.911
History of perianal surgery (%)	49 (48.0%)	117 (41.2%)	166 (43.0%)	0.232
Location, *n* (%)				0.917
Ileal	18 (18.9%)	51 (18.7%)	69 (18.8%)	
Colonic	14 (14.7%)	39 (14.3%)	53 (14.4%)	
Ileocolonic	63 (66.3%)	183 (67.0%)	246 (66.8%)	
Upper gastrointestinal	7 (6.9%)	22 (7.7%)	29 (7.5%)	0.772
Behavior, *n* (%)				**0.001**
B1 (inflammatory)	66 (64.7%)	141 (49.6%)	207 (53.6%)	
B2 (stricturing)	31 (30.4%)	97 (34.2%)	128 (33.2%)	
B3 (penetrating)	5 (4.9%)	46 (16.2%)	51 (13.2%)	
Perianal disease, *n* (%)	46 (45.1%)	163 (57.0%)	208 (53.9%)	**0.038**
Severity of malnutrition				**<0.001**
Moderate		141 (36.5%)		
Severe		143 (37.0%)		
NRS-2002, *n* (%)				**<0.001**
< 3	87 (85.3%)	70 (24.6%)	157 (40.7%)	
≥3	15 (14.7%)	214 (75.4%)	229 (59.3%)	
Weight loss, *n* (%)				**<0.001**
No	102 (100%)	12 (4.3%)		
> 5% within past 6 months		156 (55.5%)		
> 10% beyond 6 months		113 (40.2%)		
Low BMI (kg/m^2^), *n* (%)				**<0.001**
No	102 (100%)	111 (39.2%)		
< 18.5		77 (27.2%)		
< 17		95 (33.6%)		
Inflammatory load, *n* (%)	61 (67.0%)	203 (77.8%)		**0.042**
CRP (mg/L)	34.4 ± 41.6	43.5 ± 46.6	41.0 ± 45.4	0.085
PLT (10^9^/L)	311.9 ± 122.7	370.6 ± 146.2	356.1 ± 142.8	**<0.001**
Hb (g/L)	122.6 ± 26.3	112.7 ± 26.3	115.1 ± 26.6	**0.040**
Leukocyte count (10^9^/L)	7.1 ± 2.4	7.3 ± 2.7	7.3 ± 2.7	0.408
Neutrophil count (10^9^/L)	4.9 ± 2.1	5.5 ± 4.9	5.4 ± 4.3	0.116
ESR (mm/H)	25.8 ± 21.1	29.3 ± 22.7	28.4 ± 22.3	0.177
ALB (g/L)	40.4 ± 8.5	37.7 ± 8.7	38.3 ± 8.7	**0.015**
PA (g/L)	196.3 ± 77.2	173.2 ± 75.5	178.8 ± 76.5	**0.021**
Creatinine (mmol/L)	68.9 ± 18.1	62.4 ± 19.4	64.0 ± 19.3	**0.006**
Uric acid (umol/L)	343.7 ± 95.3	306.4 ± 104.8	316.0 ± 103.5	**0.002**
Vitamin D (ng/mL)	16.7 ± 11.3	14.4 ± 10.4	15.0 ± 10.7	0.108

ALB, Albumin; BMI, body mass index; CDAI, Crohn’s disease activity index score; CRP, C-reactive protein; ESR, erythrocyte sedimentation rate; Hb, hemoglobin; PLT, platelet; PA, prealbumin; Y, year. Bold-faced values indicate statistically significant at alpha < 0.05.

### Prevalence of malnutrition in CD patients

In our present study, the majority of CD patients (298, 77.4%) were identified as nutritional risk. According to simplified GLIM criteria for diagnosing malnutrition, 284 participants (73.6%) were diagnosed with malnutrition, while 102 participants (26.4%) were classified as non-malnutrition (Table 1). Among the malnourished CD patients, 141 cases (36.5%) were categorized as moderate malnutrition, and 143 cases (35.4%) were identified as severe malnutrition. The average BMI was 18.2 ± 2.3 kg/m^2^ and the mean CDAI score was 122.9 ± 70.5 in malnourished CD patients (Table 1). Interestingly, 269 malnourished patients (60.8%) exhibited obvious involuntary weight loss, even though their BMI was ≥18.5 kg/m^2^. Furthermore, 172 patients had a BMI lower than 18.5 kg/m^2^, representing 60.6% of malnourished individuals and 44.6% of the total patients.

**Figure 2:**
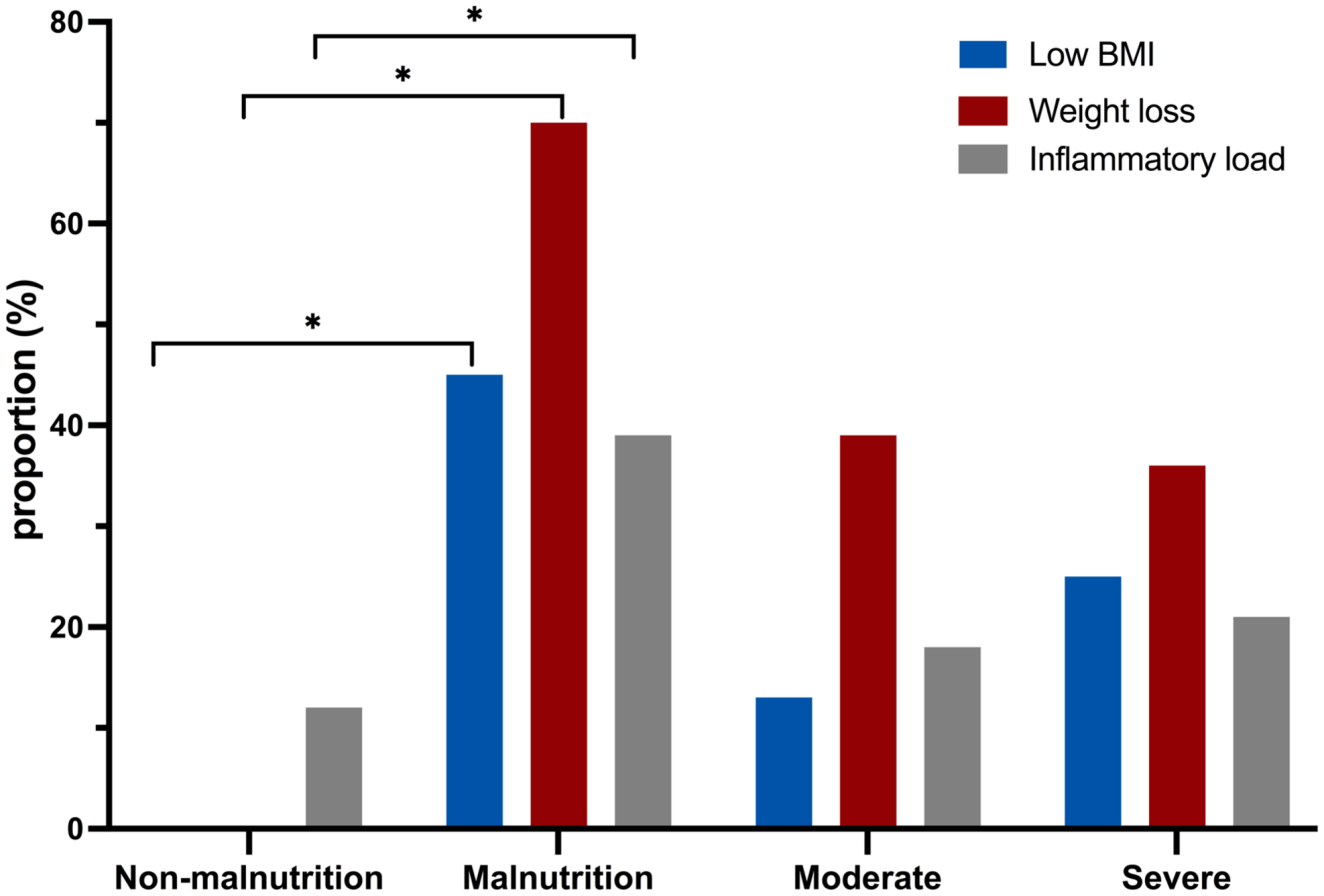
The comparison of laboratory indicators between malnourished and non-malnourished patients using the GLIM assessment. *p < 0.05.

The specific phenotypic and etiologic criteria of simplified GLIM in this study are depicted in Figure 2. The results indicated that weight loss was the most common phenotypic criterion for diagnosing malnutrition according to the simplified GLIM criteria. Additionally, it was observed that CD patients classified as moderately or severely malnourished had significantly lower BMI and weight loss compared to non-malnourished patients.

There were no significant differences in gender, disease location, smoking history, and intestinal surgery history between patients with malnutrition and those without malnutrition. Patients in the malnutrition group were younger at the age of assessment (28.3 ± 10.3 years vs. 33.9 ± 12.5 years, *p* < 0.001), onset (21.6 ± 9.2 years vs. 24.9 ± 12.6 years, *p* = 0.024) and diagnosis (24.7 ± 9.3 years vs. 29.5 ± 11.8 years, *p* = 0.001) compared to those in the non-malnutrition group (Table 1). Patients were classified into B1-B3 phenotypes based on disease behavior according to the Montreal classification (22). The majority (143, 50.4%) of CD patients with malnutrition were classified as B2 or B3 phenotype (*p* = 0.001). Furthermore, CD patients who were malnourished exhibited a higher incidence of perianal lesions in comparison to subjects without malnutrition (*p* < 0.038) (Table 1).

### The relationship between malnutrition and laboratory parameters in patients with CD

**Figure 3:**
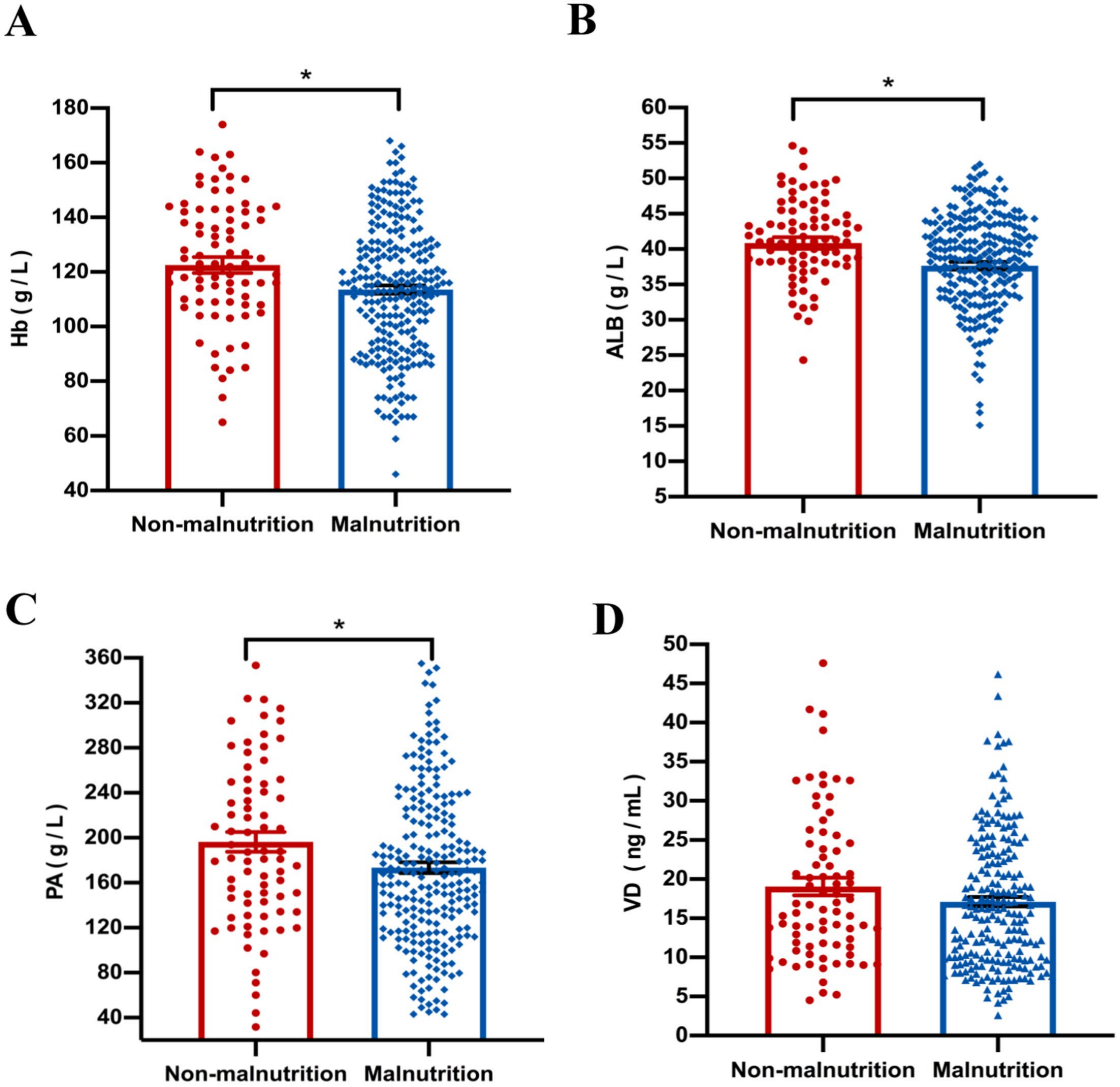
The comparison of nutritional indicators, including Hb **(A)**, ALB **(B)**, PA **(C)** and VD **(D)**, was conducted between malnourished and non-malnourished patients. *p < 0.05. Hb, hemoglobin; ALB, albumin; PA, prealbumin; VD, Vitamin D.

**Figure 4:**
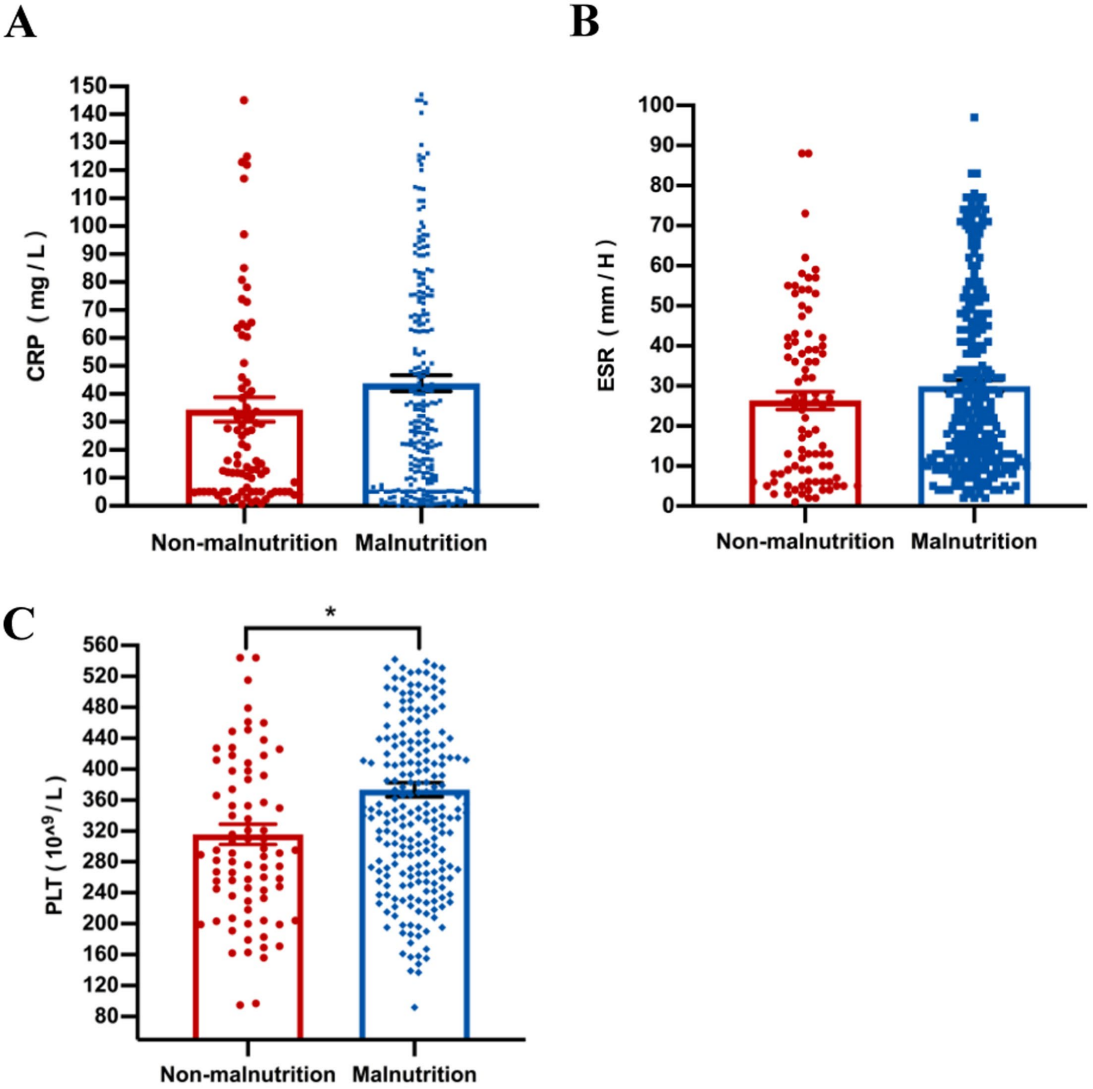
The comparison of laboratory inflammatory, including CRP **(A)**, ESR **(B)**, and PLT **(C)**, was conducted between malnourished and non-malnourished patients. *p < 0.05. CRP, C-reactive protein; ESR, erythrocyte sedimentation rate; PLT, platelet.

Our results showed that compared with the non-malnutrition group, the levels of some nutritional indicators including hemoglobin (*p* = 0.040), albumin (*p* = 0.015) and prealbumin (*p* = 0.021) were significantly lower in the malnutrition group; however, there was no difference of vitamin D levels between the two groups (Figure 3). Furthermore, the levels of platelet (*p* < 0.001) were higher in patients with malnutrition; however, there were no differences of other parameters, including CRP and ESR, between the two groups, as shown in Figure 4.

### Univariate and multivariate analysis of clinical characteristics and nutritional status in relation to endoscopic remission

The median follow-up time was 15.2 weeks (ranged by 14–20 weeks). Results of univariate logistic regression analysis showed that significant differences were observed in hemoglobin (*p* = 0.011), albumin (*p* = 0.009) and prealbumin (*p* = 0.001), which exhibited positive associations with endoscopic remission (Table 2). Moreover, we also found that patients with weight loss (>10%) (*p* = 0.006), low BMI (<17 m^2^/kg) (*p* = 0.020), malnutrition (*p* = 0.009), and severe malnutrition (*p* = 0.002) had negatively associated with endoscopic remission. Variables that demonstrated statistical significance in univariate analysis or are clinically recognized were subsequently subjected to multivariate logistic regression. The results showed that malnutrition, as assessed by simplified GLIM, independently influences endoscopic remission (OR 0.195 CI 0.044–0.874, *p* = 0.033). In addition, we also found that duration of disease (OR 0.865, CI 0.764–0.978, *p* = 0.021), CRP (OR 1.010, CI 1.002–1.014, *p* = 0.014) and prealbumin (OR 1.008, CI 1.002–1.014, *p* = 0.014) were independent factors influencing endoscopic remission of CD patients after controlling for potential confounding variables.

**Table 2:** Univariate and multivariate analysis of clinical characteristics and nutritional status in relation to endoscopic remission.

	Univariate analysis		Multivariate analysis	
	Odds ratio (95% CI)	*P*	Odds ratio (95% CI)	*P*
Male, *n* (%)	1.356 (0.875, 2.101)	0.173		
Duration of disease	0.935 (0.856, 1.021)	0.133	0.865 (0.764, 0.978)	**0.021**
BMI, kg/m^2^	1.040 (0.971, 1.115)	0.263	0.869 (0.736, 1.026)	0.098
CDAI	0.998 (0.992, 1.003)	0.412	0.996 (0.991, 1.001)	0.154
History of intestinal surgery				
Yes	0.996 (0.643, 1.543)	0.987	0.930 (0.546, 1.587)	0.791
Disease location of CD				
Ileal	Reference			
Colonic	0.905 (0.437, 1.876)	0.789		
Ileocolonic	0.898 (0.522, 1.544)	0.697		
Upper gastrointestinal	1.29 (0.60–2.76)	0.513		
Disease behavior of CD				
B1 (inflammatory)	Reference			
B2 (stricturing)	0.684 (0.432, 1.082)	0.105		
B3 (penetrating)	0.872 (0.466, 1.632)	0.699		
Perianal disease				
Yes	1.173 (0.777, 1.769)	0.447		
Malnutrition				
Yes	0.543 (0.344, 0.859)	**0.009**	0.195 (0.044, 0.874)	**0.033**
Severity of malnutrition				
Moderate	Reference			
Severe	0.634 (0.388, 1.035)	0.068		
Weight loss				
No	Reference			
>5%	0.805 (0.492, 1.316)	0.387	2.840 (0.682, 11.825)	0.151
>10%	0.457 (0.262, 0.796)	**0.006**	1.548 (0.365, 6.565)	0.553
Low BMI, kg/m^2^				
No	Reference			
<18.5	1.314 (0.778, 2.218)	0.307	2.330 (1.222, 4.441)	**0.010**
<17	0.535 (0.316, 0.907)	**0.020**	1.144 (0.575, 2.279)	0.702
Inflammatory load				
No	Reference			
Yes	0.849 (0.548, 1.315)	0.463	0.903 (0.570, 1.433)	0.666
CRP (mg/L)	0.998 (0.993, 1.002)	0.335	1.010 (1.002, 1.014)	**0.014**
PLT (10^^9^/L)	0.999 (0.997, 1.000)	0.093		
Hb (g/L)	1.012 (1.003, 1.021)	**0.011**	0.996 (0.982–1.011)	0.600
Leukocyte count (10^9^/L)	0.966 (0.890, 1.049)	0416		
Neutrophil count (10^9^/L)	0.931 (0.847, 1.024)	0.142		
ESR (mm/H)	0.987 (0.977, 0.998)	**0.017**	0.994 (0.977–1.011)	0.480
ALB (g/L)	1.042 (1.010, 1.075)	**0.009**	1.032 (0.977, 1.089)	0.262
PA (g/L)	1.005 (1.002, 1.008)	**0.001**	1.008 (1.002, 1.014)	**0.014**
Vitamin D (ng/ml)	1.011 (0.987, 1.037)	0.369		
NRS2002				
<3	Reference			
≥3	0.833 (0.558–1.242)	0.370	1.039 (0.640, 1.688)	0.876

ALB, Albumin; BMI, body mass index; CDAI, Crohn’s disease activity index score; CRP, C-reactive protein; ESR, erythrocyte sedimentation rate; Hb, hemoglobin; PLT, platelet; PA, prealbumin; Y, year. Bold-faced values indicate statistically significant at alpha < 0.05.

## Discussion

Malnutrition is common among patients with CD and has been demonstrated to impact their prognosis (29). It is vital to identify malnutrition in IBD patients. The GLIM criteria were developed to establish a global consensus on malnutrition diagnosis and has been validated in patients with some diseases (8, 9). However, there are very limited studies reporting its applicability in CD patients (11, 12). In particular, there is a lack of the application of GLIM criteria in IBD patients in China. In this study, we assessed malnutrition using the simplified GLIM criteria in a primary cohort of 386 CD patients. Our results revealed a malnutrition prevalence of 73.6% among CD patients, with 36.5% classified as moderate malnutrition and 37% as severe malnutrition. Furthermore, our current study demonstrated that disease behavior, age of onset, and CDAI were associated with nutritional status in patients with CD. Hence, employing the simplified GLIM criteria may be considered advantageous in making clinical decisions for the therapy of CD patients.

The prevalence of malnutrition appears to exceed the range of 20 to 40% reported in earlier studies (30–32). Previous studies have shown that GLIM criteria had a higher detection rate of malnutrition compared to ESPEN criteria; however, this analysis only included 53 surgical IBD patients (12). Another study reported an incidence of malnutrition of 69.5% in patients with CD; the simplified GLIM criteria used in this study were generally consistent with the ESPEN, SGA, and WHO criteria (33). This inconsistency may be attributed to two next reasons: (1) the lack of consensus on the exact criteria for assessment of malnutrition in CD patients; (2) our cases were affected by active diseases, age, and other factors; (3) moreover, patients receiving biologic treatment may be more likely to experience comorbid malnutrition.

Our research demonstrates significant differences in disease status between malnourished and non-malnourished patients. Malnourished patients exhibited lower levels of albumin and hemoglobin, while displayed elevated platelet counts. This finding is crucial for understanding the nutritional status of patients with Crohn’s disease and for guiding clinical management. Patients in the malnutrition group were found to be younger than those in the non-malnutrition group, regardless of their age at assessment, onset, or diagnosis. This was consistent with prior studies indicating that an earlier onset of symptoms could result in heightened malnutrition and inflammation. Moreover, our study found that CD patients classified as B2 and B3 according to the Montreal classification of disease behavior had a higher risk of malnutrition. This may be due to the presence of intestinal fistulas with varying degrees of narrowing or obstruction, which not only increased energy expenditure but also impaired nutrient digestion and absorption (29). In addition, we also revealed that various indicators, including platelet, hemoglobin, albumin, creatinine, and uric acid, were associated with nutritional status. This may be, at least in part, due to the fact that inflammatory reactions in the intestines of CD patients leads to the release of inflammatory mediators, which stimulates the bone marrow to produce more platelets, resulting in elevated platelet levels (34, 35). Additionally, CD causes intestinal inflammation and ulcers, which impairs digestive and absorptive functions. Prolonged inflammation and malnutrition also affect protein synthesis and metabolism, potentially causing decreased levels of substances such as hemoglobin, albumin, and creatinine (29, 36).

Currently, the treatment goal for CD is endoscopic remission. However, the clinical correlation between simplified GLIM defined malnutrition and endoscopic remission rates in patients with CD remains unclear. In our current study, CD patients were classified into three groups using simplified GLIM criteria: no malnutrition, moderate malnutrition, and severe malnutrition. Our findings indicated that patients with malnutrition exhibited a decreased rate of endoscopic remission. Conversely, patients with severe malnutrition encountered greater challenges in attaining endoscopic remission. We also found that patients who were initially malnourished had a lower rate of achieving endoscopic remission; moreover, patients with severe malnutrition may face greater challenges in attaining endoscopic remission. It should be noted that results of multivariate analysis showed a significant correlation between malnutrition, CRP, and prealbumin with endoscopic remission. This indicated that simplified GLIM defined malnutrition has demonstrated its ability to predict endoscopic remission rates in CD patients. However, there are some disadvantages to the GLIM criteria in patients with IBD. For instance, the degree of inflammation in IBD patients varies, which can result in differing levels of malnutrition severity. Additionally, patients with IBD rarely assess their muscle condition during routine practice, which raises uncertainty regarding the applicability of alternative muscle indicators.

This study has several limitations. First, this study involves a retrospective analysis of an observational cohort. The design may restrict our analytical capabilities, and the data obtained from a single center might not adequately represent the entire population of patients with CD. Second, the follow-up period is relatively short to evaluate the observed results. Longer follow-up duration is necessary to assess the correlation between simplified GLIM and clinical outcomes. Besides, nutritional therapy may significantly influence patient outcomes. Future studies should perform stratified analyses to elucidate its specific effects on this patient group. Additionally, the absence of data on decreased muscle mass and other laboratory indicators in the original database could impact the GLIM assessment. Thus, Future research should include the collection of detailed data on muscle loss and long-term tracking of patients’ nutritional status to comprehensively determine the relationship between nutritional status and prognosis of CD patients.

In conclusion, this study’s primary findings indicate that malnutrition identified using the simplified GLIM is associated with age, CDAI, behavior, hemoglobin, and albumin, providing prognostic value for endoscopic remission in CD patients. Future long-term follow-up studies on CD patients assessed using the simplified GLIM criteria should be conducted to evaluate the impact of improved nutritional status on quality of life and disease prognosis. Additionally, multicenter clinical studies should be initiated to validate the effectiveness and feasibility of this nutritional assessment tool in CD patients.

## Data Availability

The raw data supporting the conclusions of this article will be made available by the authors, without undue reservation.
